# Skin Regeneration in Diabetic Rats Using Gold Nanoparticles–Bioactive Glass Oil-in-Water Cream

**DOI:** 10.3390/ma19112276

**Published:** 2026-05-27

**Authors:** Sorin Marian Mârza, Robert Cristian Purdoiu, Adrian Valentin Potârniche, Mariana Tătaru, Cosmin Peştean, Andras-Laszlo Nagy, Alexandru Flaviu Tăbăran, Sidonia Gog-Bogdan, Ionel Papuc, Mirela Moldovan, Zsejke-Réka Tóth, Lucian Baia, Klara Magyari

**Affiliations:** 1 Faculty of Veterinary Medicine, University of Agricultural Sciences and Veterinary Medicine, 400372 Cluj-Napoca, Romania; sorin.marza@usamvcluj.ro (S.M.M.); robert.purdoiu@usamvcluj.ro (R.C.P.); adrian.potarniche@usamvcluj.ro (A.V.P.); mariana.tataru@usamvcluj.ro (M.T.); cosmin.pestean@usamvcluj.ro (C.P.); alexandru.tabaran@usamvcluj.ro (A.F.T.); sidonia.bogdan@usamvcluj.ro (S.G.-B.); 2 Department of Biomedical Sciences, Ross University School of Veterinary Medicine, Basseterre P.O. Box 334, Saint Kitts and Nevis; nagyandras26@gmail.com; 3 Faculty of Pharmacy, Iuliu Haţieganu University of Medicine and Pharmacy, 400012 Cluj-Napoca, Romania; mmoldovan@umfcluj.ro; 4 Interdisciplinary Research Institute on Bio-Nano-Science, Babes-Bolyai University, 400271 Cluj-Napoca, Romania; zsejke.toth@ubbcluj.ro (Z.-R.T.); klara.magyari@ubbcluj.ro (K.M.); 5 Faculty of Physics, Babes-Bolyai University, 400084 Cluj-Napoca, Romania; 6 Institute for Research-Development-Innovation in Applied Natural Sciences, Babes-Bolyai University, 400294 Cluj-Napoca, Romania; 7 INSPIRE Research Platform, Babes-Bolyai University, 400084 Cluj-Napoca, Romania

**Keywords:** diabetic wound, tissue regeneration, bioactive glasses, gold nanoparticles

## Abstract

Diabetes is a chronic disease that severely impairs wound healing, slowing wound closure; thus, the risk of infection and increases the occurrence of other complications. The development of a suitable material that can accelerate the process of chronic wound regeneration, particularly in diabetic wounds, remains a significant challenge. In the present study, Sepigel 305^®^ paraffin-based oil-in-water cream containing spherical gold nanoparticles–bioactive glass was used in rats with induced diabetes mellitus. After wound closure, the stage of regeneration was evaluated histopathologically. It was shown that the wounds treated with the experimental product were closed macroscopically after 14 days, but the histological images still indicated an inflammatory process, suggesting incomplete deep dermal healing. Macroscopic closure of wounds treated with the studied cream after 14 days, which is a normal time for skin healing, represents a successful outcome in diabetic patients because the risk of bacterial infection is reduced, and thus the chance of complete healing increases.

## 1. Introduction

Wound healing is an important process that occurs when the skin or other tissues are damaged. In individuals with diabetes, this process is often impaired due to hyperglycemia and other complications, leading to delayed wound healing and increased risk of infection. Diabetes affects insulin production and utilization, and chronic hyperglycemia damages small blood vessels and nerves, including those in the skin [1]. This vascular and neural damage reduces tissue regeneration and wound repair. Factors such as wound size, depth, patient age, general health, infection, and poor circulation can further influence the healing rate [1].

Effective treatment of diabetic wounds typically involves a combination of approaches, including wound care, infection control, and blood sugar management. Medications, specialized dressings, and other medical interventions are used to promote healing and prevent complications [2,3]. However, current treatments—such as debridement, drainage, growth factor therapies, shockwave therapy, and hyperbaric oxygen therapy—have significant limitations in addressing the multifactorial nature of diabetic wounds [2,3].

Recent research has focused on innovative strategies to overcome these limitations. Bioactive peptides can stimulate fibroblast and keratinocyte migration, enhance angiogenesis, and improve extracellular matrix synthesis, significantly accelerating wound healing [4,5]. Plant extracts such as *Rhus coriaria* [6], *Ginkgo biloba* extract [7], or *Symphytum officinale* extract [8] have demonstrated potent wound-healing properties. Additionally, polymer composites loaded with antioxidants such as epigallocatechin-3-gallate [9] or quercetin [10] can inhibit oxidative stress and inflammation, facilitating healing.

Advanced biomaterials, particularly bioactive glasses, have been widely applied from bone regeneration to drug delivery to soft tissue regeneration [11,12,13,14]. Modifying silicate glass matrices with various components allows their properties to be tuned. Thus, the bioactive glass compositions can enhance the M1-to-M2 macrophage switch, accelerating the wound-healing process [15], and the composite can be modified to promote angiogenesis, facilitating the wound-healing process [16,17]. It was demonstrated that introducing spherical gold nanoparticles into the silicate glass enhances human keratinocyte proliferation [18,19]. Embedding these gold nanoparticle-bioactive glass composites in Vaseline ointments improved wound healing in healthy rats [20,21]. Despite these advances, current therapeutic strategies for diabetic wounds present several limitations. Debridement and drainage are primarily supportive and do not actively stimulate tissue regeneration. Growth factor therapies often suffer from rapid degradation and limited bioavailability. Hyperbaric oxygen therapy is costly and not widely accessible, while shockwave therapy shows variable clinical outcomes. Moreover, many existing approaches fail to simultaneously address key pathological features of diabetic wounds, including chronic inflammation, impaired angiogenesis, and oxidative stress.

Wound healing is achieved through four phases: hemostasis, inflammation, proliferation, and remodeling [22]. The first stage is hemostasis, which begins immediately after injury, and the formation of a clot occurs. After the bleeding is controlled, the inflammatory stage begins with the migration of inflammatory cells [23]. The proliferation stage starts within the first 48 h and continues for up to 14 days from the injury. In this stage, the wound closes as a result of angiogenesis, fibroplasia, and re-epithelialization [24]. The remodeling phase begins in two to three weeks, and it can last a year or more. In this final stage, the organism tries to recover the normal structure and function of the tissue. In diabetic wounds, excessive inflammation, reduced angiogenesis, and decreased fibroblast proliferation impair regeneration [1,25]. The average healing time of a diabetic foot ulcer is 112 days, although in favorable cases it may be as short as 30 days [26].

On this basis, and continuing the previous work, for diabetic wounds, we incorporated bioactive glass with spherical gold nanoparticles (BGAuSP) into an oil-in-water (O/W) cream, structurally characterized it, and applied it to diabetic rat wounds. Accordingly, it was monitored wound closure in diabetic rats, followed by histopathological evaluation. Bacteriological testing was conducted to exclude pathogenic contamination.

To place this work within the current state of the art, a comparative overview of recent studies using bioactive glass, gold nanoparticles, and composite biomaterials for diabetic wound healing is presented in Table 1. These studies consistently demonstrate accelerated wound closure through mechanisms such as angiogenesis, modulation of inflammation, and stimulation of fibroblast activity. However, most approaches rely on hydrogels or complex delivery systems, while simpler topical formulations remain less explored, especially in diabetic models.

In this context, the present study proposes a simplified, yet multifunctional topical formulation based on gold nanoparticles–bioactive glass embedded in an oil-in-water cream. Unlike more complex delivery systems, such as hydrogels or scaffolds, this formulation is easy to apply, combining the bioactivity of ionic release with nanoparticle-mediated cellular stimulation. Furthermore, the study extends previous findings obtained in healthy models to a diabetic wound model and integrates both macroscopic and histopathological evaluation, providing a more comprehensive assessment of tissue regeneration.

## 2. Materials and Methods

### 2.1. Materials

Hydrogen tetrachloroaurate (III) hydrate (HAuCl_4_·3H_2_O, 99.99%, Sigma-Aldrich, Burlington, MA, USA), trisodium citrate dihydrate (ACS, 99.0%, Merck, Darmstadt, Germany), and Pluronic F127 (powder, BioReagent, suitable for cell culture, Sigma-Aldrich, Schnelldorf, Germany) were used for gold nanoparticles (AuSP) synthesis.

Tetraethyl orthosilicate (TEOS, ≥99%, Merck, Darmstadt, Germany), calcium nitrate tetrahydrate (Ca(NO_3_)_2_∙4H_2_O, ≥99%, Lach-Ner, Neratovice, Czech Republic), and triethyl phosphate (TEP, ≥99%, Merck, Darmstadt, Germany) were used for the bioactive glass (BG) synthesis. Nitric acid (HNO_3_, 65%, Nordic Chemical, Cluj-Napoca, Romania) was used for hydrolysis.

Ingredients used for the cream preparation: caprylic/capric triglycerides (Croda, Snaith, UK), paraffin oil, cetearyl alcohol (Vitamar, Bucharest, Romania), Sepigel 305^®^ (polyacrylamide & C13-14 Isoparaffin & laureth-7, Seppic, Paris, France), Euxyl PE 9010^®^ (phenoxyethanol & ethylhexylglycerin, Schülke & Mayr, Norderstedt, Germany), glycerin, rice powder (*Oryza sativa* starch, Elemental, Oradea, Romania), and distilled water. Phosphate-buffered saline (PBS, Sigma-Aldrich) was used for in vitro assays. Ultrapure water and absolute ethanol were used throughout the whole experimental process. All chemicals were used as received without further purification.

To induce diabetes, streptozotocin (STZ, Sigma-Aldrich, Germany) and nicotinamide (NA) were used.

The surgery required the following materials: biopsy punch with a diameter of 5 mm (by relaxing the skin the excision has a diameter of 6 mm); antisepsis solutions: Lifo Scrub (Braun Medical, Melsungen, Germany) and 70% ethanol; scalpel with fixed blade; surgical tweezers and hemostatic tweezers; scissors; non-absorbable needles and sutures (Nyllion 4.0); clipper; depilatory cream; sterile dressing; elastic bandage and superglue. For the positive control, cream with silver sulfadiazine (AgSD, Regen-Ag, Fiterman Farma, Iasi, Romania) was used. One gram of the cream contains 10 mg of silver sulfadiazine, with excipients including macrogol 6-stearate, glycol stearate, macrogol 32-stearate, cetostearyl alcohol, liquid paraffin, macrogol cetostearyl ether 12, propylene glycol, methyl parahydroxybenzoate (E218), propyl parahydroxybenzoate (E216), and purified water.

### 2.2. Cream Preparation and Characterization

An oil-in-water (O/W) cream was prepared to incorporate the gold nanoparticles–bioactive glass (BGAuSP), which represented the active ingredient of the formulation. For the preparation of the gold nanoparticles (AuSP) and BGAuSP, the synthesis described in the previous study was used [19,21,29]. For details of their synthesis and structural properties, see Supplementary Materials (Figures S1 and S2).

The cream was prepared as an oil-in-water disperse system by heating the aqueous and oily phases separately, followed by phase mixing under vigorous stirring. First, the aqueous phase was prepared by mixing distilled water at a controlled temperature (50 °C ± 2 °C) together with Sepigel 305^®^, glycerol, and the preservative Euxyl PE 9010^®^. Sepigel 305^®^ is a multifunctional vehicle with thickening, stabilizing, texturizing, and tissue-adhering properties, and creates a gel texture when mixed with water [30]. The oil phase was prepared separately by melting the cetearyl alcohol together with paraffin oil in a water bath at 60 ± 2 °C. Caprylic/capric triglycerides were added after removing the previous melted mixture from the water bath, and then this lipophilic phase was added to the aqueous phase, heated at the same temperature, under continuous stirring. Cream homogenization continued until it reached room temperature, then rice powder (6%) and the BGAuSP (18%) were homogeneously incorporated into the cream composition under stirring. The rice powder was added to absorb wound fluids and exudates, and rice starch may also reduce the inflammation [31].

A cone & plate rheometer (CAP 2000+, Brookfield, Middleboro, MA, USA) was used to determine the viscosity of the products tested (BGAuSP-O/W and AgSD creams). The following viscometer settings were established: hold time 10 s, run time 30 s, spindle 08. The rheological parameters: viscosity, shear rate, and shear stress were recorded at different rotation speeds varying from 5 to 50 rpm. Measurements were performed in triplicate, at 23 °C ± 1 °C; the mean values of determined parameters ± standard deviation were reported.

The creams were structurally characterized using X-ray diffraction (XRD) and UV-Vis spectroscopy. and Fourier Transform Infrared (FT-IR). The XRD patterns were recorded with the Shimadzu XRD 6000 diffractometer (Kyoto, Japan) using CuKα radiation (λ = 1.54 Å) with a Ni filter, in a 2θ range from 10° to 80° with a speed of 2°/min. The FT-IR absorption spectra were recorded in reflection configuration with a Jasco FT-IR 6200 spectrometer (Jasco, Tokyo, Japan), at room temperature, in the range 400–4000 cm^−1^; spectral resolution of 4 cm^−1^; using the well-known KBr pellet technique. The UV-Vis spectra were recorded using a JASCO-V650 spectrophotometer equipped with an ILV-724 integration sphere (Wien, Austria) using BaSO_4_ as a reference, with a spectral range of 190–800 nm.

The in vitro assay of O/W and BGAuSP-O/W creams was performed by immersion in phosphate-buffered saline (PBS) at a concentration of 10 mg∙mL^−1^ for 24 h at 37 °C. The cream was placed in the dialysis membrane (por 4, MWCO 12,000-14,000). The evaluation of the changes in the cream after immersion in PBS was followed by XRD and FT-IR.

### 2.3. In Vivo Assays

#### 2.3.1. Animal Care and Use

The biological material used in the experiment consisted of adult rats with induced diabetes. Rats included in the study were adult female rats, weighing 150–180 g of the species laboratory rat, family *Muridae*, *Wistar-Lewis* line [32]. The choice of rats as an experimental model was based on the following criteria: high docility during experimental handling, reduced susceptibility to bacterial infection and spontaneous tumor development, and good adaptability to captive housing conditions. Furthermore, the use of female animals contributes to improved reproducibility by minimizing variability related to stress-induced hormonal fluctuations and injury-related confounders frequently reported in male cohorts. The rats were purchased from the Experimental Medicine Centre of the Iuliu Hațieganu University of Medicine and Pharmacy, Cluj-Napoca, Romania. After purchase, the rats were transferred to and housed at the Unit of Reproduction and Use of Laboratory Animals of the Faculty of Veterinary Medicine, Cluj-Napoca, Romania, where the experiment was conducted. The 30 adult female rats included in the study were randomly assigned to two groups (n = 15 per group) using a simple randomization process: each rat was assigned a number, and numbers were drawn randomly to allocate animals to the experimental or control group, ensuring an unbiased distribution of potential confounding factors. The sample size of 15 rats per group was determined based on statistical power analysis. Using an estimated effect size derived from previous pilot studies we performed with the BGAuSP-O/W cream (effect size f = 0.8), an alpha level of 0.05, and a desired power of 0.8 (80%), the minimum required sample size was calculated to be 12 rats per group. It included 15 rats per group to account for potential dropouts and ensure robust statistical analysis. This randomization and sample size justification support the reliability and reproducibility of the in vivo results.

All rats in the study received standard maintenance and feeding conditions: temperature of 23 °C, relative humidity of 55%, and 12 h light/dark cycles, according to ISO 10993-2 [33]. They were fed with standard granulated food for rodents and had access to water ad libitum. The experiment was approved by the Bioethics Committee of the University of Agricultural Sciences and Veterinary Medicine Cluj-Napoca, no. 237/01.02.2021, and authorized by the Sanitary Veterinary and Food Safety Directorate, Cluj-Napoca, by Project Authorization no. 253/07.04.2021.

#### 2.3.2. Experimental Induction of Diabetes

Diabetes mellitus was induced in rats using the streptozotocin-nicotinamide (STZ-NA) protocol based on its extensive validation as a reliable experimental model of type 2 diabetes mellitus [34,35]. First, all the animals were weighed (±1 g) and fasted for 6 h before the start of the experiment. Blood was drawn from the tail vein of each Wistar rat, and the fasting blood glucose levels were determined immediately before the experiment (day 0), using a commercial blood glucose meter (Accu-Chek^®^ Active, Roche, Germany). The rats received a single intraperitoneal injection of STZ (65 mg/kg b.w., dissolved in 0.1 mM citrate buffer, pH 4.5), 15 min after NA (100 mg/kg b.w., dissolved in 0.9% saline solution) [36]. The rats were returned to their cages and provided with normal food and 10% sucrose water, which was replaced after 24 h with regular water. After 7 days of STZ-NA administration, the diabetic status of the rats was evaluated. The fasting blood glucose levels were measured from blood samples collected from the tail vein. The animals exhibiting blood glucose levels of more than 150 mg/dL were considered diabetic and used in the present study [37]. Prado et al. [38] reported that in STZ-diabetic mice, following the onset of hyperglycemia, there is a decrease in cellular density in both the epidermis and dermis, delayed maturation of collagen fibers, and increased oxidative stress in the skin. Additionally, Andrade et al. [39] show that after only fifteen days of induced diabetes, there is increased inflammation, oxidative stress, reduced vessel number, and decreased expression of VEGF, eNOS, and TGF-β1. Thus, we decided that after 7 days of STZ-NA administration, the surgical procedure to assess wound healing should be initiated.

#### 2.3.3. Surgical Procedure

The protocol consisted of performing two dermal excisions in the dorsal region of the withers on either side of the spine. Before surgery, the rats were weighed and then anesthetized with a mixture of Ketamine (Ketamine Narkamon Bio, Bioveta, Czech Republic, 60 mg/kg) and Xylazine (Xylazin Bio 2%, Bioveta, Czech Republic, 6 mg/kg). The anesthetic mixture was injected intraperitoneally, and the dose for each rat was calculated. After anesthesia, the rats were prepared for surgery. The skin excision site was the dorsal region of the thorax, from the T1 vertebra to the L1 vertebra, with the starting point in the region of the withers extending towards the pelvis. After trimming, a hair-removal product was applied to ensure complete depilation. To avoid burns, the depilatory cream was left in contact with the skin for only 3 min instead of the recommended 5 min, after which it was removed with a special plastic scraper. First, the area was thoroughly cleaned with sterile swabs soaked in Lifo Scrub (Braun Medical). Then, 70% alcohol swabs were used to ensure effective antisepsis. In rats, the well-developed panniculus carnosus muscle causes a rapid apposition of the wound edges, which will modify the rate of healing and skin regeneration in spontaneous injuries. To counteract this effect and to follow the skin regeneration phenomenon in stages, it was necessary to use 1 mm thick semi-rigid silicate splints, which were fixed to the skin around the excision and which were previously disinfected. The splints were obtained by cutting out silicate disks with a diameter of 15 mm using a metal punch. These disks were further cut inside with a 6 mm metal punch, creating a silicate ring with a total diameter of 15 mm and a hole diameter of 6 mm. For immediate fixation of the silica ring, superglue was applied to the side in direct contact with the skin, which allowed subsequent fixation of the rings with non-absorbable sutures, so that each silica ring was fixed to the skin with four symmetrical sutures [40].

To perform the dermal excisions, the following steps were performed: the rats were restrained in lateral recumbency, after which the skin in the dorsal region, along the line of the spine, was clamped and pulled with two hemostatic clamps, thus forming a skinfold. Using the biopsy punch, fixed at a distance of about 8 mm from the edge of the skinfold, four excisions were performed, which were obtained by gently rotating the biopsy punch through both layers of the skinfold. The distance between the two excisions was approximately 16 mm as a result of the skinfold unraveling [40]. After performing the skin excisions, they were cleaned with a sterile swab, after which silica rings were applied.

The experimental and commercial products were applied according to the following working protocol: the rats were restrained in a sterno-abdominal position, with their hind limbs facing the researcher, so that the dorsal region with the two excisions remained free, providing direct access for cream applications. On the left side, for the first batch, on the left skin excision, the BGAuSP-O/W cream was applied, and on the right side, no treatment was applied, and the excision served as a control; for the second batch, on the left skin excision, the AgSD was applied, and on the right side no treatment was applied, and the excision served as a control. For each excision, 100 μL of cream was applied topically. Applications were performed on days 4 and 7 after surgery, following the same protocol and concentration for each administration. This timing strategy was selected to intervene during periods of maximal cellular responsiveness, particularly targeting impaired angiogenesis and fibroblast activation characteristic of diabetic wound healing. After each application, the wound was covered with sterile elastic bandages to prevent infection. Fifteen days after wound closure, the rats were euthanized by anesthetic overdose and cervical dislocation. From the site of each skin excision, where the creams were applied, and the healing process occurred, samples of skin tissue were taken, covering both the site where the scar formed and approximately 0.5 cm of surrounding normal tissue for histopathological examination.

#### 2.3.4. Bacteriological Test

Samples were collected on the first day of surgery, before creation of the skin defects. Samples were collected using sterile swabs after the hair in the area of excision was trimmed. After collection, samples were seeded in Petri dishes on blood-enriched agar and incubated at 37 °C for 24 h.

To reduce human intervention and for accurate and rapid identification of Staphylococcus species, the Vitek^®^ 2 Compact (bioMérieux, Marcy l’Etoile, France) was used. The working steps were as follows: standardization, dilution, and traceability, followed by transfer of preliminary results. The instrument manages all steps automatically. After primary isolation, handling is minimized in a simple inoculum preparation. Standardized 0.5–0.6 µL McFarland inoculum was inserted into the cassette, then entered into the computer software via barcode, with a sample identification number. The identification was performed automatically. The identification process of *Staphylococcus* species took between 2 h and 8 h [41].

#### 2.3.5. Measurements of Wound Size Reduction

Images were evaluated, and free, open-source software was used (ImageJ software, version 1.53a). Each image was transformed into an 8-bit, grayscale image, to 336 × 336 pixels, to ensure homogeneity. Before taking pictures, a ruler was used to measure the wound; the calibration value was 27 pixels/mm. A freehand ROI was traced to include the wound, and the changes in the ROI were empirically evaluated. To have a graphical representation of the wound, for each ROI traced, a histogram was obtained. The same process was used for all products and pictures obtained. Empirical evaluation of the ROI was necessary because the statistical evaluation of the ROI changes over time was not significant.

#### 2.3.6. Histological and Immunohistochemical Methods

At the end of the experimental period, the animals were euthanized by cervical dislocation, and skin samples were harvested for histological examination. Skin samples were fixed in 10% buffered neutral formalin and embedded in paraffin, sections were made at 4 micrometers, and the slides were stained by Hematoxylin–Eosin (HE) and Masson’s trichrome methods. The histological sections were examined under an Olympus BX 51 microscope, and images were taken using the Olympus UC 30 digital camera (Olympus Corporation, Tokyo, Japan); they were processed using an image acquisition and processing program: Olympus Stream Basic (Olympus Corporation, Tokyo, Japan).

An immunohistochemical examination was performed using a mouse monoclonal anti α-SMA antibody (clone 1A4) (Abcam ab76549) in a 1:800 dilution by a Leica Bond-Max (Leica Microsystems, Wetzlar, Germany) automated immunostainer using a polymer-based detection system (Leica Biosystems, Wetzlar, Germany) with 3,3′-diaminobenzidine (DAB) as the chromogen.

#### 2.3.7. Statistical Analysis

To assess the significance of differences among groups, a one-way analysis of variance (ANOVA) for each parameter at specified time intervals was used. In using ANOVA, we assumed normality and homogeneity of variance criteria. Significant ANOVA outcomes prompted subsequent analyses using Tukey’s Honest Significant Difference (HSD) post hoc test to pinpoint particular group discrepancies. Statistical significance was at *p* < 0.05 in all cases.

## 3. Results

### 3.1. Cream Characterization

**Figure 1:**
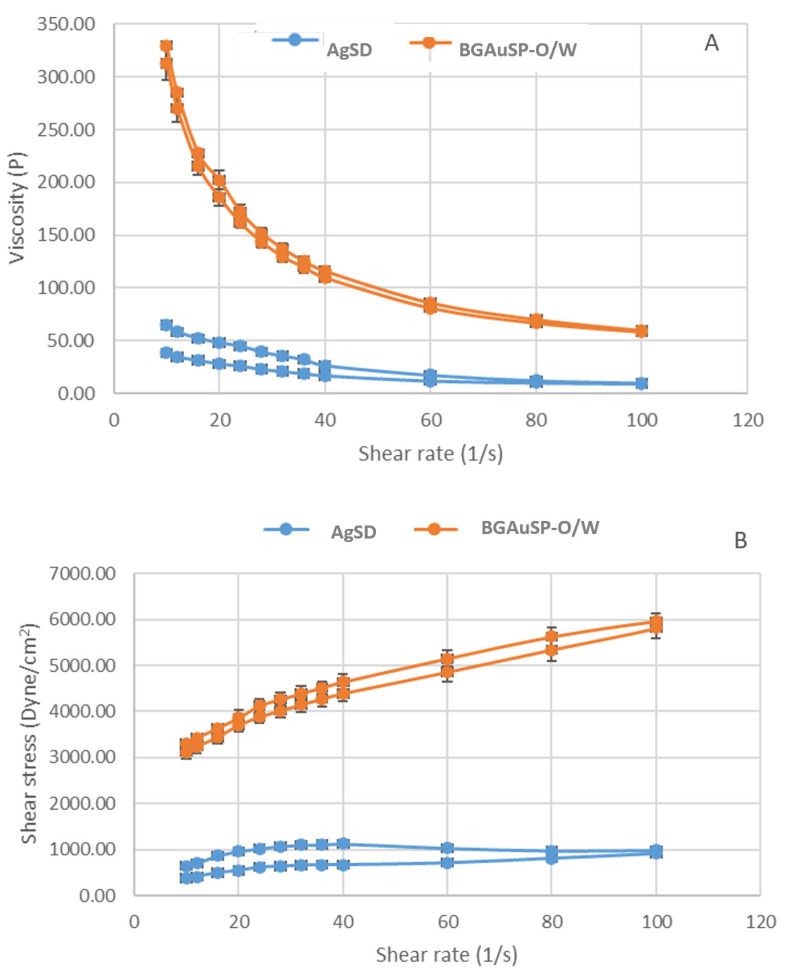
Flow curve (**A**) and rheogram (**B**) of the AgSD (blue lines) and BGAuSP-O/W (orange lines) creams (mean values ± SD).

The rheological behavior of the gels was analyzed by plotting the viscosity values as a function of the shear rate (Figure 1). The AgSD cream is less viscous; its viscosity values vary from 65.00 ± 2.50 P at 5 rpm to 9.83 ± 0.52 P at 50 rpm, while for BGAuSP-O/W cream, the viscosity varied from 330.00 ± 2.50 P at 5 rpm to 59.58 ± 1.81 P at 50 rpm. The ability of the products to retain their shape can be assessed from the values of the thixotropic index, which is calculated from viscosity values measured at two shear rates, one increased by a factor of 10, as the ratio between the viscosity at low shear value and the viscosity at high shear value. The AgSD has a thixotropic index of 6.61, while BGAuSP-O/W cream has a thixotropic index of 5.54.

The thixotropic index values indicate that both formulations exhibit high levels of time-dependent structural breakdown and recovery, consistent with structured semisolid systems. Both values fall within the high-thixotropy range (when thixotropic index values of semisolid pharmaceutical systems are greater than 5), specific to formulations with an internal structure that undergoes significant reversible breakdown under shear but rapidly rebuilds upon removal of stress. This behavior is advantageous for topical wound-care formulations, enabling low-resistance during dispensing and spreading, followed by a rapid structural recovery after application to ensure retention at the application site. The higher thixotropic index of AgSD indicates a more pronounced structural rearrangement during shear, a faster transition from a semisolid state to a flowable state; the slightly lower thixotropic index of BGAuSP-O/W reflects a more stable internal network that resists structural disruption to a greater extent. These results suggest that both products have desirable rheological behaviors for topical formulations designed to balance ease of application with retention at the application site.

The rheograms of the two tested creams are presented in Figure 1B, where the shear stress increases as the shear rate increases. The analysis of the plots was performed to fit a mathematical model. For AgSD, the curve had a better fit (confidence fit of 94.1%) with the Casson model, which characterizes a non-Newtonian material. This model enabled the calculation of several parameters, including plastic viscosity (1.4 P) and yield stress (322 D/cm^2^). The BGAuSP-O/W plot analysis showed a good fit to the Bingham model, with a confidence fit of 96.6%; therefore, it behaves like a viscoplastic material, rigid at low stress and a viscous fluid at high stress. The value of plastic viscosity was 28 P, and the calculated yield stress was 3086 Dyne/cm^2^.

**Figure 2:**
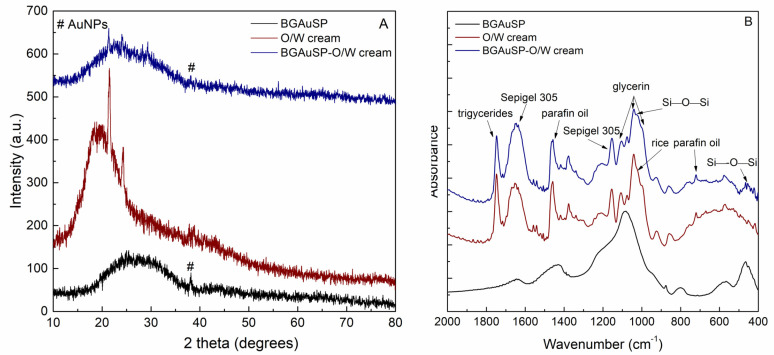
XRD patterns (**A**) and FT-IR spectra (**B**) of the BGAuSP (black lines), O/W-cream (red lines), and BGAuSP-O/W cream (blue lines).

The XRD pattern of the cream displayed predominantly amorphous characteristics with two distinct diffraction peaks at 2θ~21.8° and 24.2°, which can be assigned to the structural features of petrolatum (Figure 2A) [41]. The presence of AuSP in the BG and cream is indicated by the reflection of gold (COD 00-900-8463) at 2θ~38.2° [19,42]. After incorporation of the BGAuSP in the O/W cream, the broad signal of the O/W cream slightly shifted to a lower value, suggesting the bond between the BGAuSP and the O/W cream.

The FT-IR spectrum of the O/W presents the spectral features of ingredients (Figure 2B). The band at 1747 cm^−1^ can be assigned to C = O stretching of carbonyl ester from caprylic/capric triglycerides [43]. The bands at 1648 and 1156 cm^−1^ originated from the stretching vibration of C = O groups and deformation vibrations of the CH_2_ groups from polyacrylamide [44], which is the main component of Sepigel 305^®^. The absorption bands at 1100 and 1038 cm^−1^ can be assigned to the C-O stretching vibration of glycerin [45]. Another characteristic absorption band of glycerin can be identified at 993 cm^−1^ and assigned to the skeletal backbone vibration coupled with the C-O stretch [45]. The presence of paraffin oil is represented by the bands at 1457 and 720 cm^−1^ that are assigned to C-H bending and CH_2_ rocking vibrations, respectively [46]. By adding the BGAuSP to the O/W cream, the typical signal of the silicate network appears at 456 cm^−1^ and is assigned to the Si-O-Si bending mode, while the shoulder at 1020 cm^−1^ is attributed to the Si-O-Si stretching vibration [47].

**Figure 3:**
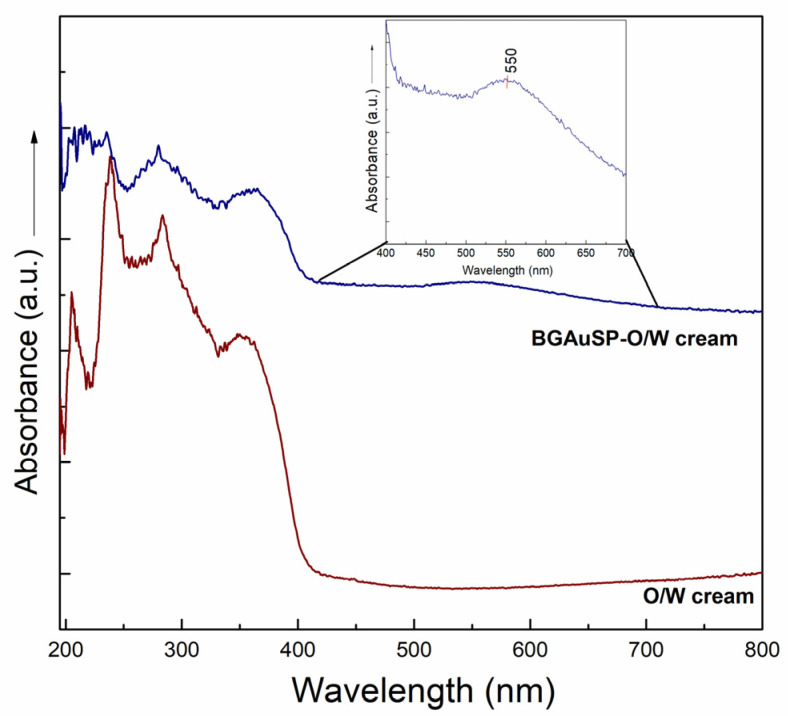
UV-Vis spectra of O/W (red line) and BGAuSP-O/W creams (blue line).

The presence of AuSP in the BGAuSP-O/W cream was demonstrated by UV-Vis spectroscopy (Figure 3). In addition to the cream, ingredient bands between 190 and 400 nm were seen. Thus, a broad band at about 550 nm appeared, indicating the presence of polydisperse gold nanoparticles with an average diameter of about 70 nm.

**Figure 4:**
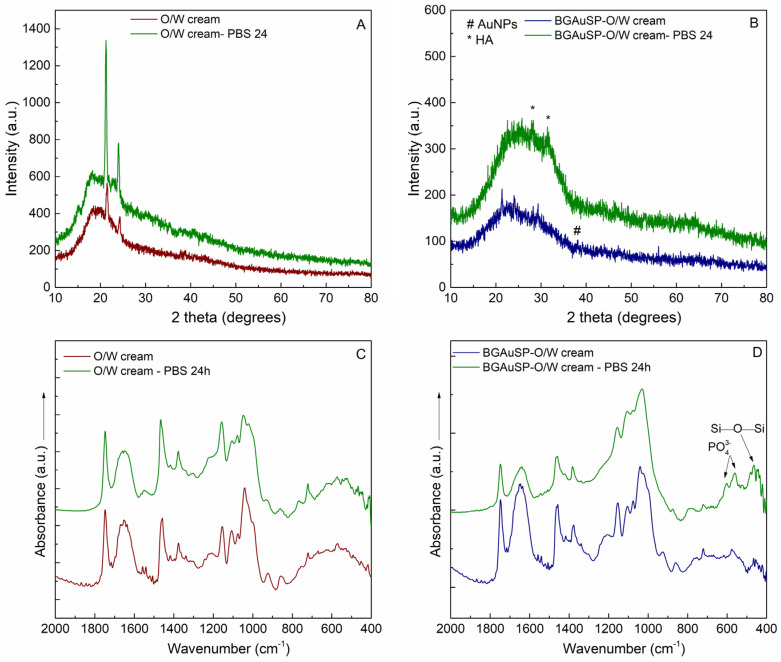
XRD patterns (**A**,**B**) and FT-IR spectra (**C**,**D**) of the O/W (**A**,**C**) and BGAuSP-O/W-cream (**B**,**D**) before and after immersion in PBS for 24 h.

To simulate the interaction between the cream and tissue, the creams were immersed in PBS for one day at a temperature of 37 °C. The dialysis membrane was used to allow the solution to pass through while preventing the cream from leaking out. After 24 h, the XRD pattern of the O/W cream became more crystalline, and the XRD pattern of the BGAuSP-O/W cream showed the specific reflections of the apatite crystalline phase, evidenced by the reflection at 2θ = 28° and 31.5° (Figure 4A,B). The FT-IR spectrum confirms these results in the case of the BGAuSP-O/W cream; the P-O bending vibrations appear at 602 and 557 cm^−1^ after immersion in PBS (Figure 4D) [47].

### 3.2. Skin Regeneration Potential of Cream

#### 3.2.1. Bacteriological Assays

**Figure 5:**
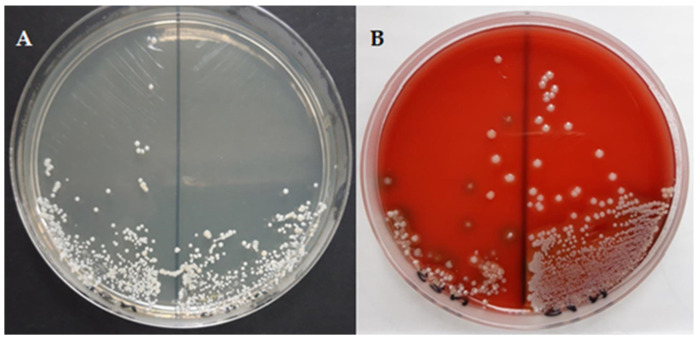
Representative macroscopic images of bacterial colonies grown on glucose-enriched agar (**A**) and on blood agar (**B**).

Pathogenic bacterial microflora can influence the process of skin healing and regeneration. A bacteriological analysis was conducted to detect skin bacterial load, as microbial contamination can hinder healing, cause inflammation, and reduce the efficacy of regenerative therapies. Macroscopic examination revealed that round, smooth, small-to-medium-sized non-hemolytic colonies, arranged in grape-like clusters and pigmented in creamy/chalky white and orange, developed on the seeding medium (Figure 5A,B). Comparison of the two culture media was performed to optimize the detection and characterization of the skin microbiota, as their distinct nutritional conditions and differential properties can influence bacterial growth, colony morphology, and pigment expression, allowing the identification of a broader diversity of microorganisms present in the analyzed samples.

Smears were taken from the grown colonies and stained using the Gram stain and then examined microscopically. Bacteria of the genus *Staphylococcus* were identified on microscopic examination. The genus *Staphylococcus* comprises several species and is considered to be a saprophytic skin microbiota because it colonizes the skin and mucous membranes. Most of the *Staphylococcus* species are part of the indigenous microbiota and are non-pathogenic. The most common bacterial species found on the skin are staphylococci, which are also the most important causes of nosocomial infections and associated skin infections [48]. From the genus *Staphylococcus*, the following species have been identified: *Staphylococcus lentus*, *Staphylococcus auricularis*, and *Staphylococcus xylosus*. The identification process of these species ranged from 89% to 94%, being classified with the identification message of very good. *Staphylococcus lentus* belongs to the *Staphylococcus sciuri* group along with *S. vitulinus* and is part of the Gram-positive, oxidase-positive, coagulase-negative category of the bacterial genus *Staphylococcus*, consisting of clustered cocci [49]. *Staphylococcus lentus* is a commensal bacterium that colonizes the skin of several animal species [50]. *Staphylococcus xylosus* is usually described as a common non-pathogenic rodent skin bacterium. Research on the pathogenicity of *S. xylosus* as a primary pathogen in human and veterinary medicine is scarce [51]. *Staphylococcus auricularis* belongs to the Gram-positive, oxidase-positive category of bacteria, coagulase-negative members of the bacterial genus *Staphylococcus*, consisting of clustered cocci [49]. It is a commensal bacterium that has a marked predilection for the external auditory canal [52]. In addition, the observed colony characteristics, particularly the absence of hemolytic activity and the predominance of coagulase-negative staphylococci, suggest a predominantly commensal and non-virulent microbial profile. This composition is consistent with findings reported in the literature, indicating that the normal skin microbiota may contribute to maintaining skin homeostasis and limiting colonization by pathogenic microorganisms. Although the present data do not directly demonstrate a protective effect, they support the hypothesis of a beneficial role of commensal flora in maintaining skin health.

#### 3.2.2. Evaluation of Wound Closure

To comprehensively capture the critical stages of the diabetic wound-healing model, we selected days 4, 7, 12, and 14 as key sampling points. Day 4 represents the transition from early inflammation toward the beginning of proliferative responses, including initial immune cell infiltration and onset of angiogenesis and granulation formation [53]. Day 7 corresponds primarily to the inflammatory and early proliferative phases, where immune cell migration, nascent vascular sprouting, and early matrix deposition can be assessed; these processes are particularly important in diabetic models because inflammation persists and delays progression [53]. Day 14 typically represents the peak of the proliferative phase, when fibroblast activity, collagen deposition, and neovascularization are most active; in diabetic rodents, these events are markedly delayed and altered relative to healthy controls, making this time point informative for evaluating the effects of therapeutic interventions [52,53,54]. Compared to the known protocol, the day on which the wound was almost closed was also selected (day 12).

**Figure 6:**
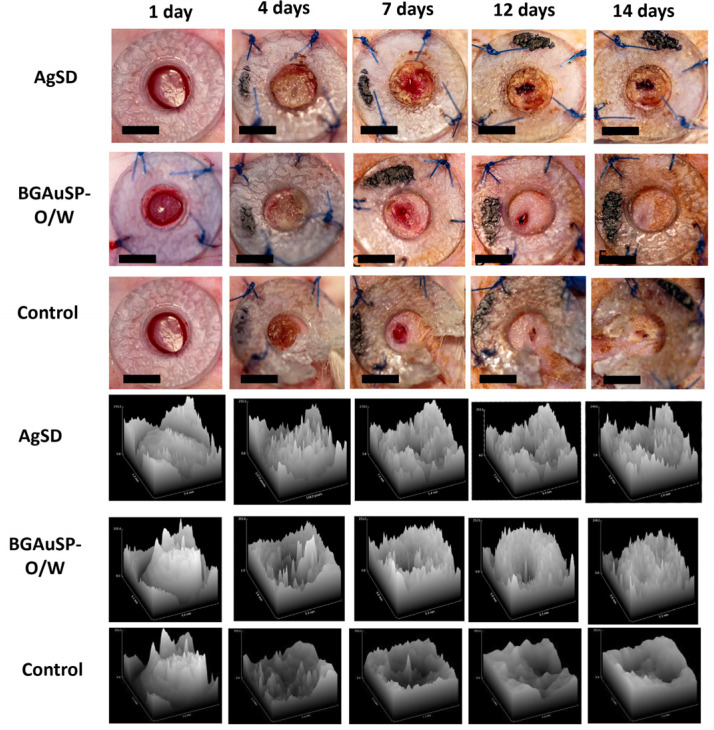
Full-thickness skin defects in rats and the evolution of wound healing treated with AgSD and BGAuSP-O/W cream and wound healing without treatment (control) in 3D at 1, 4, 7, 12, and 14 days after surgery. Scale bar: 5 mm.

**Figure 7:**
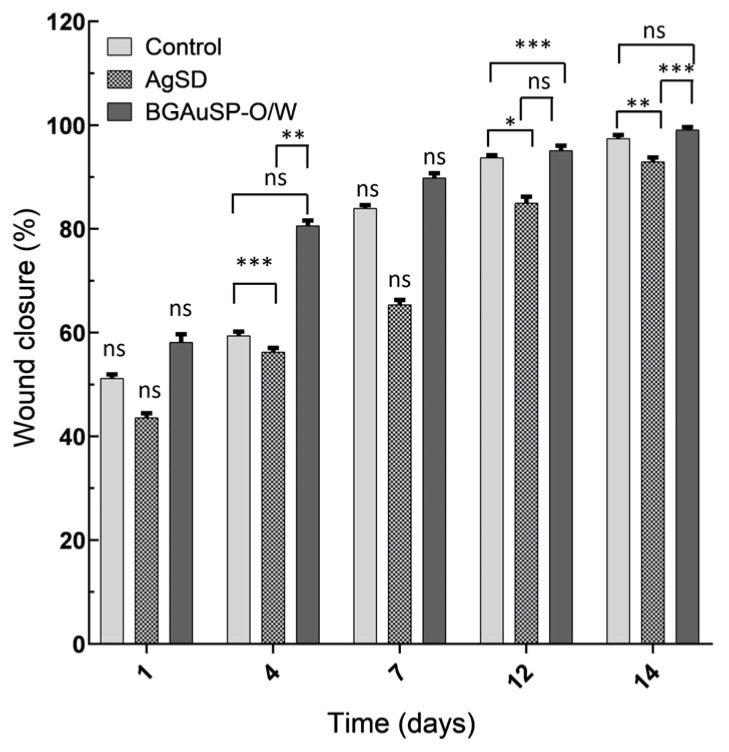
Percentage of wound closure for the defect treated with AgSD and BGAuSP-O/W cream and wound healing without treatment (control). ns = statistically insignificant, * *p* < 0.05; ** *p* < 0.01; *** *p* < 0.001.

Thus, the wounds were photographed on days 1, 4, 7, 12, and 14 post-surgery to quantify the degree of healing (Figure 6). The wound treated with BGAuSP-O/W cream was completely healed at 14 days compared with the AgSD case, where the wounds still had a small crust even at 14 days (Figure 7). Wound closure progressed over time in all groups; however, treated groups demonstrated accelerated healing compared to controls. By day 14, wounds treated with BGAuSP-O/W cream achieved near-complete closure, while AgSD-treated wounds showed minor residual crust formation. Quantitative analysis confirmed significant improvements in wound-healing parameters for both treatments compared to controls at multiple time points (*p* < 0.05). Overall, BGAuSP-O/W cream demonstrated faster macroscopic wound closure, while AgSD exhibited stronger effects on selected statistical parameters at intermediate stages.

For comparative purposes, statistical analysis was performed. Quantitative analysis of wound-healing parameters revealed no significant differences among groups at baseline (day 1) for any measured metric, including wound area, perimeter, mean gray value, or distribution parameters (ANOVA, all *p* > 0.05; e.g., Area: *p* = 0.842; Mean: *p* = 0.916), confirming comparable initial wound conditions. On day 7, the analysis revealed no significant differences among the groups for any measured metrics (all *p* > 0.05), whereas on the 4th, 12th, and 14th days, notable findings included significant differences in Mean, Median, and Kurtosis, necessitating further examination via Tukey’s HSD to identify specific group differences. On day 4, significant group effects were observed for Mean (ANOVA, *p* = 0.021), Median (*p* = 0.034), and Kurtosis (*p* = 0.041). Post hoc Tukey’s HSD analysis indicated significant differences between the control and AgSD groups (Mean: *p* = 0.018; Median: *p* = 0.027) and between the control and BGAuSP-O/W groups (Mean: *p* = 0.022; Median: *p* = 0.031). On day 12, ANOVA revealed significant differences for Mean (*p* = 0.009), Median (*p* = 0.014), and Kurtosis (*p* = 0.036), with post hoc comparisons again showing significant differences between the control group and both treatment groups (control vs. AgSD: *p* = 0.011–0.028; control vs. BGAuSP-O/W: *p* = 0.013–0.033). Similar trends were observed on day 14, where Mean (*p* = 0.006), Median (*p* = 0.010), and Kurtosis (*p* = 0.029) differed significantly among groups. Thus, specific group contrasts on days 4, 12, and 14, particularly for Mean and Median, indicated significant differences primarily between the control and AgSD groups and between the control and BGAuSP-O/W groups, highlighting the varying impacts of the treatments on the central tendency of wound measurements. Within-group analysis demonstrated a progressive reduction in wound area and perimeter over time in all groups (*p* < 0.05 for time effect), consistent with wound closure. Overall, both AgSD and BGAuSP-O/W treatments resulted in significantly improved wound-healing metrics compared with untreated controls, with AgSD showing a stronger effect on selected parameters at specific time points. Although the study demonstrated statistical significance, it did not delve into effect sizes or practical significance. Future research should consider these aspects, alongside exploring underlying mechanisms and broader applications through larger trials.

#### 3.2.3. Histopathological Evaluation of the Skin Defect

**Figure 8:**
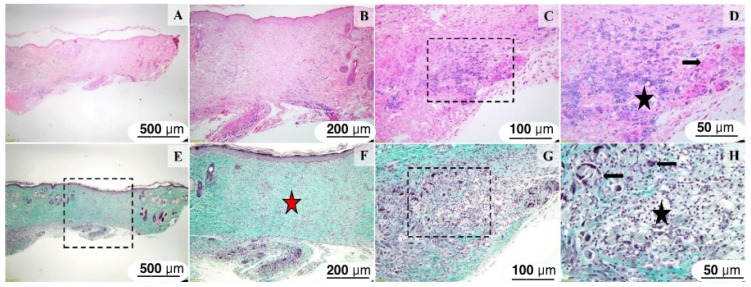
Histological images of the skin and subcutis from the animals of the BGAuSP-O/W group. The intact, mildly hyperplastic epidermis (**B**,**F**) is supported by a moderately fibrous dermis (red star, (**F**)), devoid of pilosebaceous units. Multifocally, mainly within the subcutis and deep dermis, there are a few, well-demarcated granulomas, containing a few foreign-body-type giant cells (black arrows, (**D**,**H**)), centered on an amorphous, basophilic foreign material (black star, (**D**,**H**)). H&E stain (images (**A**–**D**)) and Masson’s trichrome (images (**E**–**H**)). Scale bars: (**A**,**E**) 500 μm (50× magnification); (**B**,**F**) 200 μm (100× magnification); (**C**,**G**) 100 μm (20× magnification) and (**D**,**H**) 50 μm (400× magnification).

The BGAuSP-O/W group exhibited a continuous, moderately hyperplastic epidermis supported by granulation tissue replacing the dermis and subcutis, with moderate lymphocytic infiltration and focal granulomatous inflammation. In contrast, the AgSD-treated group showed more pronounced dermal fibrosis extending into the hypodermis, with a less prominent inflammatory component. The control group displayed incomplete re-epithelialization, discontinuous epidermis, and marked fibrotic remodeling with reduced vascularization. Histological evaluation of the sections from the group treated with BGAuSP-O/W indicated that the surgical wound is partly healed, and the epidermis is continuous and moderately hyperplastic. The superficial and deep dermis were replaced with excess granulation tissue, infiltrated by numerous lymphocytes. Pilosebaceous units were absent in the wound area (Figure 8B,F), occasionally within the deeper dermis and hypodermis. A focal, mild-to-marked, granulomatous inflammation associated with the presence of numerous macrophages and giant cells (arrows) centered on amorphous, basophilic, compact-to-coarse granular foreign material (interpreted as the tested article) was observed. The panniculus carnosus is absent in the section (Figure 8A,B,E,F).

**Figure 9:**
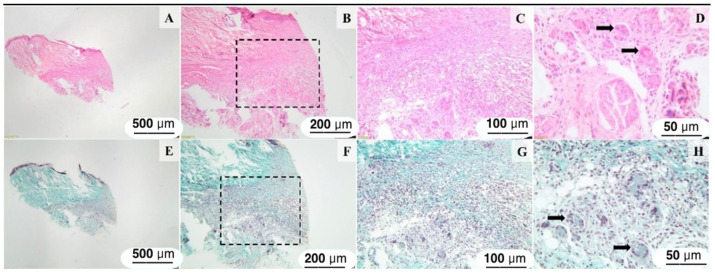
Histological images of the skin and subcutaneous tissue from the animals of the AgSD group. Intact hyperplastic epidermis (**B**), dermal fibrosis, and mild granulomatous reaction in the deep dermis and hypodermis, containing few foreign-body-type giant cells (arrows) are observed (**B**–**D**,**F**–**H**). H&E stain (images (**A**–**D**)) and Masson’s trichrome (images (**E**–**H**)). Scale bars: (**A**,**E**) 500 μm (50× magnification); (**B**,**F**) 200 μm (100× magnification); (**C**,**G**) 100 μm (20× magnification), and (**D**,**H**) 50 μm (400× magnification).

Histological sections of the skin and subcutis from the AgSD-treated group showed a wound surface covered by a mildly hyperplastic epidermis. There is mild to marked dermal fibrosis extending from the superficial dermis through the subcutis. Within the deep dermis and hypodermis, a well-demarcated granulomatous reaction consisting of numerous macrophages, a few foreign-body-type giant cells (arrows), and lymphocytes was present (Figure 9D).

**Figure 10:**
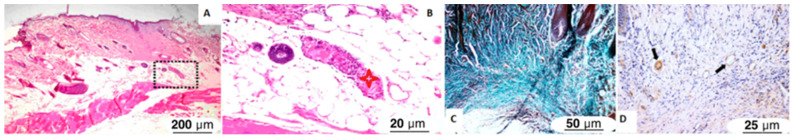
Histopathological images of the skin and subcutaneous tissue from the animals of the control groups showing (**A**,**B**) extending from the superficial dermis through the subcutis, there is marked dermal fibrosis (red star); focally, especially in the deep dermis and subcutis, the fibrous connective tissue contains many lymphocytes and macrophages interspersed with many blood vessels ((**D**), arrows). The epidermis is focally ulcerated (with perilesional epidermal focal acanthosis), and covered by a thick serocellular crust. H&E (images (**A**,**B**)), TM stain (images (**C**)), and α-SMA immunolabeling (**D**). Scale bars: (**A**) 200 μm (100× magnification); (**B**) 20 μm (200× magnification); (**C**) 50 μm (400× magnification), and (**D**) 25 μm (200× magnification).

Histological sections of the skin and subcutis from the control excision revealed a discontinuous epidermis (Figure 10A), and mild to marked dermal fibrosis extending from the superficial dermis through the subcutis, with partial loss of the skin appendages (Figure 10B. The fibrous proliferation, consisting of reactive fibroblasts admixed with fibrocytes, few blood vessels (Figure 10D), and abundant collagenous extracellular matrix infiltrated the deep dermis and subcutis. (Figure 10B—red star and Figure 10D arrow).

## 4. Discussion

### 4.1. Characterization and In Vitro Performance of BGAuSP-O/W Cream

The viscosity of topical products is both a quality and an efficacy criterion, as it influences the products’ spreadability and the delivery of active compounds at the skin surface. For both analyzed products, namely AgSD and BGAuSP-O/W cream, shear-thinning behavior was observed, as their viscosity decreases when the shear rate values increase. Shear-thinning behavior is associated with the thixotropic behavior, which facilitates the skin application of the product, as it becomes thinner when a force is applied and then recovers the initial state when the applied force is removed, thus helping it remain in place. The higher viscosity of BGAuSP-O/W cream is provided by the polyacrylamide from Sepigel 305^®^ composition, which also ensures a more rapid return to the initial state after skin application. The thixotropic index indicates how quickly the viscosity changes in the dispensing region; both products have a high thixotropic index, which confirms that both products keep their shape after application, which is important for a wound-healing product, to ensure that it stays at the application site. The yield stress value indicates the force that is needed for a product to flow and may be correlated with the ease of product removal from the tube.

The results obtained by the XRD, FT-IR, and UV-Vis spectroscopy indicate the bond between the BGAuSP and the O/W cream. The cream ingredients and the gold nanoparticles are present in the obtained samples, confirming the successful synthesis. Simulating in vitro behavior, it can obtain valuable information about the behavior of the tested material. It was used PBS, as it has the same salt concentration as most biological fluids. After immersion of the BGAuSP-O/W cream in the PBS, the apatite crystalline phase is present, which indicates that the cream retained the bioactive character of the introduced glass.

### 4.2. In Vivo Performance of BGAuSP-O/W Cream

The commensal bacterial species that make up the skin microbiota play an essential role in ensuring skin homeostasis and immune competence. Recent research suggests that coagulase-negative staphylococci found on the skin may stimulate the skin’s immune system to limit the colonization potential of invaders and may compete directly with invaders by producing antimicrobial molecules or by signaling antagonism [48]. In summary, it can be concluded that no pathogenic bacterial microflora was identified on the skin, and thus, it is unlikely that this microflora influenced the healing and regeneration process. The bacterial flora detected consisted of commensal species, which appeared to play a protective role in maintaining skin health.

Application of the creams to the rat wounds confirms the in vitro assays regarding the yield stress; the AgSD and BGAuSP-O/W cream could be easily applied to the skin. AgSD can be more easily squeezed from the tube, while BGAuSP-O/W cream holds better in place after skin application. This is also confirmed by the area of the hysteresis loop observed in Figure 1A; the smaller hysteresis area is correlated to a faster system recovery.

By evaluating the wound closure, the statistical analysis underscored the significant impacts of the AgSD and BGAuSP-O/W cream on wound-healing metrics, with the AgSD product consistently showing notable effects on the central tendency of wound measurements at various stages. This underscores the need for further research to fully understand the practical significance and clinical implications of these findings. However, wound closure is more rapid in the case of BGAuSP-O/W cream treatments than in the case of AgSD (Figure 7). This difference probably appeared due to the silver content of the AgSD. We obtained the same difference in a previous study for the samples with silver content, which slowed wound healing in the first week, but reduced the possibility of infection with pathogenic bacteria [21]. Faster wound closure with BGAuSP-O/W cream may be caused by the release of Ca^2+^ ions [55]. The in vitro assays show that after PBS immersion, the apatite crystalline phase is present on the BGAuSP-W/O cream surface, indicating that the cream can cover all apatite-forming steps. It is known that in the first stage, the Ca^2+^ ions from BG are exchanged with H^+^, H_3_O^+^ from solution, and the calcium content in the solution increases during the first hours [5,56]. Navarro-Requena et al. [57] show that calcium-releasing materials are potential biostimulators to be applied in dressings for chronic wound healing, as supplementation with extracellular calcium increases fibroblast metabolic activity. Ca^2+^ ions act as intracellular secondary messengers, upregulating genes related to angiogenesis (VEGF, FGF2) and extracellular matrix formation [57]. In addition to Ca^2+^, BG releases Si^4+^ ions, which stimulate fibroblast activity, collagen deposition, and endothelial cell proliferation [58,59]. AuSPs are known to promote angiogenesis, fibroblast proliferation, and keratinocyte migration via the activation of key signaling pathways, such as the TGF-β/VEGF pathway [27]. This signaling enhances vascularization and tissue remodeling, critical for diabetic wound healing. The previous in vitro assays demonstrated that BGAuSP-Vaseline cream supports keratinocyte proliferation and migration [20,21].

Histological analysis at 14 days revealed a completely regenerated epidermis in both the excisions of the batch treated with BGAuSP-O/W cream and those treated with AgSD. Regarding the dermis and hypodermis in excisions treated with BGAuSP-O/W cream, a granulomatous inflammatory reaction was observed, and dermal fibrosis was observed in excisions treated with AgSD. In the control group, the epidermis is discontinuous, and reactive fibroblasts are present in the dermis and hypodermis.

The giant cells observed near both introduced materials are multinucleated giant cells, consistent with a foreign-body giant cell reaction surrounding the applied material. The foreign-body giant cell reaction is a biological response characterized by the formation of multinucleated giant cells, which occurs when the body encounters large foreign materials, such as implants or biomaterials. After the introduction of a foreign body, macrophages migrate to the site due to various chemoattractants released during the inflammatory response. Fusion of macrophages into giant cells requires specific adhesion molecules and proteins, including DC-STAMP and E-cadherin. This fusion is a response to the persistent presence of foreign materials and is influenced by the physical and chemical properties of biomaterials. The presence of multinucleated foreign-body giant cells indicates a typical host response to exogenous materials, involving macrophage recruitment and fusion as part of the foreign-body reaction. While this process is often associated with biomaterial degradation and remodeling, the present study does not include direct evidence of material breakdown, clearance, or systemic excretion. Therefore, conclusions regarding the fate of the biomaterial should be interpreted with caution. Future studies incorporating biodistribution analysis, degradation kinetics, and systemic toxicity evaluation are necessary to fully characterize the long-term biocompatibility of the formulation.

Similar results have been obtained in recent studies, showing that gold nanocomposites and/or bioactive glasses can significantly improve diabetic wound healing. Yang et al. [27] reported a glucose-responsive multifunctional hydrogel with gold nanoclusters, which achieved over 98–99% bacterial reduction within 24 h against *S. aureus* and *E. coli* and resulted in approximately 97% wound closure within two weeks in diabetic animal models. Chang et al. [28] demonstrated that cerium-doped bioactive glass gel promotes wound healing through the bioactivity of glass, reducing inflammation, improving angiogenesis, and facilitating dermal fibroblast proliferation. A recent study showed that a multifunctional hydrogel loaded with magnesium-doped bioactive glass-induced vesicle clusters enhances diabetic wound healing by promoting intracellular delivery of extracellular vesicles [60]. A randomized controlled clinical trial showed that borate-based bioactive glass fiber matrix can accelerate the healing of diabetic foot ulcer patients [61]. These results support the validity of the obtained results, highlighting the novelty of the formulation design and the practical applicability of the simple materials used.

In summary, the proposed in vivo healing mechanism is as follows: upon application, BGAuSP-O/W cream releases Ca^2+^ and Si^4+^ ions from the BG matrix, which activate fibroblasts and endothelial cells, promoting collagen deposition and neovascularization. AuNPs further enhance wound repair by modulating inflammation and stimulating keratinocyte and fibroblast proliferation. The combination of ion release and nanoparticle-mediated cellular stimulation synergistically accelerates re-epithelialization, dermal remodeling, and vascularization, surpassing the effects of silver-based dressings (AgSD) that primarily provide antimicrobial protection but slow tissue regeneration in the early stages.

## 5. Conclusions

In conclusion, the use of BGAuSP-O/W cream has beneficial effects on wound healing and skin regeneration in rats with induced diabetes mellitus. The biomaterials induced a local cellular response characterized by the presence of macrophages and multinucleated foreign-body giant cells, suggesting active interaction between the material and host tissue. However, no direct assessment of material degradation, systemic distribution, or excretion was performed in the present study. The results show that although macroscopically the wounds were healed after BGAuSP-O/W cream treatment, the histological examination still indicates an inflammatory process, which means that at a deep cellular level, the healing is not complete, and new studies are needed to show how many days are required to achieve complete healing, and at the cellular level, rather than only macroscopically. However, when the wound is closed macroscopically, the risk of infection decreases or is eliminated. Thus, a wound closed macroscopically after 14 days can be considered a successful result. Future research should focus on elucidating the molecular and cellular mechanisms that regulate diabetic wound healing, including inflammatory processes, angiogenesis, and fibroblast activity. Additionally, analyzing the wound microenvironment, oxidative stress, and blood glucose levels, together with advanced digital and imaging-based wound monitoring, may contribute to a more precise understanding of healing dynamics. Finally, extending studies to more complex preclinical models will facilitate the development of strategies with potential clinical applications for the treatment of chronic diabetic wounds.

## 6. Patents

Klara Magyari and Lucian Baia have a patent, Porous bioactive glass doped with gold nanoparticles to be used in tissular engineering, licensed to RO132343-A2.

## Figures and Tables

**Table 1:** Comparison of selected studies on diabetic wound healing using bioactive materials and gold-based systems.

Study	Material/System	Model	Healing Time/Outcome	Key Mechanism	Comparison with the Present Study
Yang et al. [27]	Hydrogel with gold nanoclusters	Diabetic rodents	~97% closure in 14 days	Antibacterial, angiogenesis	Similar closure rate; the present system is simpler (cream)
Chang et al. [28]	Ce-doped bioactive glass gel	Diabetic model	Accelerated healing	Anti-inflammatory, angiogenesis	Comparable mechanism via ion release
Vargas Guerrero et al. [17]	Bioactive glass-based dressings (review)	Multiple	Improved healing trends	Macrophage modulation	Supports the BG mechanism used here
Mârza et al. [20,21]	BGAuSP in Vaseline	Healthy rats	Accelerated healing	Keratinocyte proliferation	The present study extends to the diabetic model
Present study	BGAuSP in O/W cream	Diabetic rats	Full closure at 14 days	Ion release, AuNP signaling	Adds diabetic context + histological insight

## Data Availability

The original contributions presented in this study are included in the article/Supplementary Material. Further inquiries can be directed to the corresponding authors.

## Supplementary Materials

The following supporting information can be downloaded at: https://www.mdpi.com/article/10.3390/ma19112276/s1, Figure S1 UV-Vis spectra (a) and TEM micrograph (b) of the AuSP and BGAuSP; Figure S2 XRD (A) and FT-IR spectra (B) of the BGAuSP. XRD pattern of BG (60SiO2·32CaO·8P2O5 mol%) and AuSP, and the FT-IR spectrum of BG are also included for comparison purposes.

## Abbreviations

The following abbreviations are used in this manuscript:

AgSD	silver sulfadiazine
AuSP	spherical gold nanoparticles
BG	bioactive glass
BGAuSP	gold nanoparticles–bioactive glass
BGAuSP-O/W	gold nanoparticles–bioactive glass in oil-in-water
O/W	oil-in-water
PBS	phosphate-buffered saline
STZ-NA	nicotinamide–streptozotocin
