# Supplementary material for: Skin Regeneration in Diabetic Rats Using Gold Nanoparticles–Bioactive Glass Oil-in-Water Cream
Source: Materials (Basel). 2026 May 27;19(11):2276. doi: 10.3390/ma19112276 (PMC13258721; doi:10.3390/ma19112276)
Supplement: Supplementary file 1 [file materials-19-02276-s001.zip › materials-4267031-supplementary.pdf]

materials

Supplementary Materials

Skin Regeneration in Diabetic Rats Using Gold Nanoparticles–Bioactive Glass Oil-in-Water Cream

Sorin Marian Mârza [1], Robert Cristian Purdoiu [1], Adrian Valentin Potârniche [1], Mariana Tătaru [1], Cosmin Peştean [1], Andras-Laszlo Nagy [2], Alexandru Flaviu Tăbăran [1], Sidonia Gog-Bogdan [1], Ionel Papuc [1,*], Mirela Moldovan [3], Zsejke-Réka Tóth [4], Lucian Baia [4,5,6,*] and Klara Magyari [4,7]

[1] Faculty of Veterinary Medicine, University of Agricultural Sciences and Veterinary Medicine, 400372 Cluj-Napoca, Romania; sorin.marza@usamvcluj.ro (S.M.M.); robert.purdoiu@usamvcluj.ro (R.C.P.); adrian.potarniche@usamvcluj.ro (A.V.P.); mariana.tataru@usamvcluj.ro (M.T.); cosmin.pestean@usamvcluj.ro (C.P.); alexandru.tabaran@usamvcluj.ro (A.F.T.); sidonia.bogdan@usamvcluj.ro (S.G-B.)
[2] Department of Biomedical Sciences, Ross University School of Veterinary Medicine, Basseterre P.O. Box 334, Saint Kitts and Nevis; nagyandras26@gmail.com
[3] Faculty of Pharmacy, Iuliu Haţieganu University of Medicine and Pharmacy, 400012 Cluj-Napoca, Romania; mmoldovan@umfcluj.ro
[4] Interdisciplinary Research Institute on Bio-Nano-Science, Babes-Bolyai University, 400271 Cluj-Napoca, Romania; zsejke.toth@ubbcluj.ro (Z.-R.T.); klara.magyari@ubbcluj.ro (K.M.)
[5] Faculty of Physics, Babes-Bolyai University, 400084 Cluj-Napoca, Romania
[6] Institute for Research-Development-Innovation in Applied Natural Sciences, Babes-Bolyai University, 400294 Cluj-Napoca, Romania
[7] INSPIRE Research Platform, Babes-Bolyai University, 400084, Cluj-Napoca, Romania
* Correspondence: ionel.papuc@usamvcluj.ro (I.P.); lucian.baia@ubbcluj.ro (L.B.)

Materials and Methods

Synthesis of gold nanoparticles–bioactive glass

The gold nanoparticles–bioactive glass (BGAuSP, $60SiO_2{\cdot}31.85CaO{\cdot}8P_2O_5{\cdot}0.15Au_2O$ mol%) was prepared by the sol–gel method. The gold amount is conventionally indicated in the oxidic form Au_2O; the gold content amount is 0.09at%.

The spherical gold nanoparticles (AuSPs) were obtained using the Turkevich–Frens method [1]. $HAuCl_4{\cdot}3H_2O$ solution was prepared (10^{-3} M). After this, the solution was heated to the boiling point of water. Afterward, $38.8\text{x}10^{-3}$M trisodium citrate solution was instantly added to the boiling gold precursor solution using a 10:1 volume ratio. The whole procedure continued for 30 min, and then it was cooled to room temperature. For further application, AuSPs were stabilized using a Pluronic F127 block copolymer solution of 0.5×10^{-3} M. The solution containing the AuSP was stirred for 20 min, followed by an aging process of 24 hours to ensure the adherence of the polymer molecules. The excess Pluronic F127 was removed by collecting the supernatant liquid from the centrifugation tubes, which were held for 30 min at 12000 rpm. The authors would like to mention that all the stabilized AuSPs were used during the preparation process of the bioactive network. Consequently, the real Au content can be considered the initial amount.

For the BGAuSP samples, the reactants were added consecutively at 1-hour intervals under continuous stirring. In the final step, the colloidal gold solution was added and stirred for 1 hour. Gelation was achieved in ≈2 days at 37°C, and the gels were aged for 3 days at 37°C. The maturated gels were dried at 110°C for 24 hours and thermally treated

 https://doi.org/10.3390/10.3390/ma19112276

at 500°C for 2 hours. All analyses were performed on powder samples, which were milled by hand using an Agate mortar. The glass samples' granulation was similar.

Methods

An FEI Technai G2 F20 high-resolution transmission electron microscope (TEM) equipped with a 200 kV, W cathode was. The samples were suspended in H_2O and dropped on a 300-mesh Cu grid. The images obtained were interpreted using ImageJ software.

Results

Structural and morphological characterization of the AuSPs and BGAuSPs

The free gold nanoparticles reveal a narrow plasmonic band in the visible region centered at 525 nm, characteristic of individual spherical gold nanoparticles, confirmed also by the TEM micrograph, which shows a small AuSP with a diameter of around 20 nm. The surface plasmon resonance bands of BGAuSP at 530 nm show the presence of nanoparticles with diameters around 25 nm, while the broadening of the absorption band indicates the polydispersity of gold nanoparticles in the glass structure (Fig. S1).

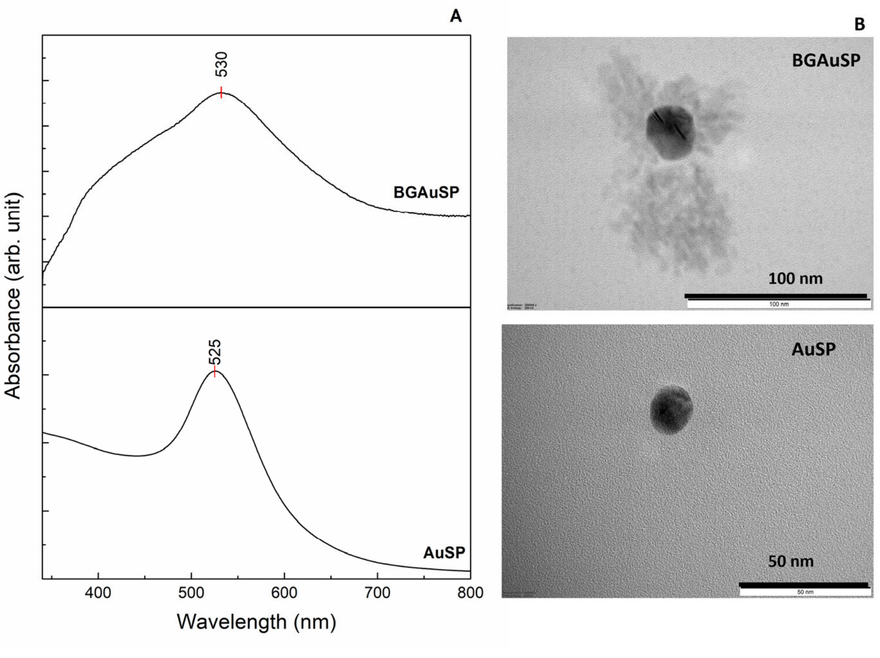

Figure S1. UV-Vis spectra (a) and TEM micrograph (b) of the AuSP and BGAuSP.

The XRD pattern of bioactive glasses revealed an amorphous structure, whereas the presence of gold nanocrystals in the glass is proven by the reflection at $2\theta=38.1°$ (Fig. 2A). The FT-IR spectra of the BG have characteristic absorption bands of silicate network, and the spectral characteristics are not influenced by the AuSP (Fig. S2) [2].

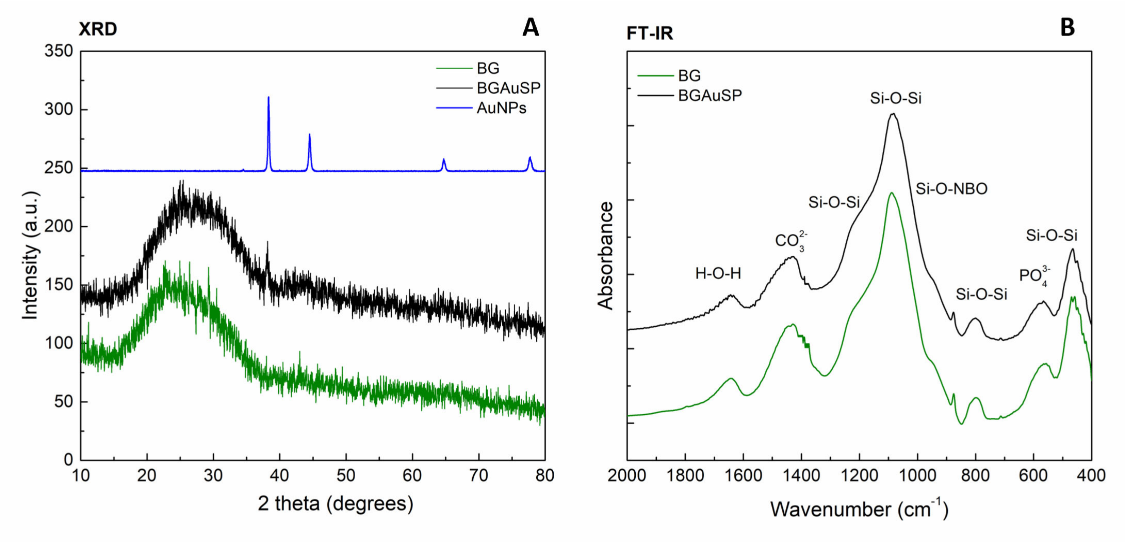

Figure S2. XRD (A) and FT-IR spectra (B) of the BGAuSP. XRD pattern of BG ($60SiO_2{\cdot}32CaO{\cdot}8P_2O_5$ mol%) and AuSP, and the FT-IR spectra of BG are also included for comparison purposes.

References

1. Frens, G. Controlled Nucleation for the Regulation of the Particle Size in Monodisperse Gold Suspensions. *Nature Physical Science volume* **1973**, *241*, 20–22.
2. Magyari, K.; Baia, L.; Vulpoi, A.; Simon, S.; Popescu, O.; Simon, V. Bioactivity Evolution of the Surface Functionalized Bioactive Glasses. *J. Biomed. Mater. Res. B Appl. Biomater.* **2015**, *103*, 261–272, doi:10.1002/jbm.b.33203.
